# Comparative genomics reveals broad genetic diversity, extensive recombination and nascent ecological adaptation in *Micrococcus luteus*

**DOI:** 10.1186/s12864-021-07432-5

**Published:** 2021-02-18

**Authors:** Yisong Li, Zhong-Zhi Sun, Jin-Cheng Rong, Bin-Bin Xie

**Affiliations:** Microbial Technology Institute and State Key Laboratory of Microbial Technology, Shandong University, Qingdao, 266237 China

**Keywords:** *Micrococcus luteus*, Population genomics, Pan-genome, Intraspecies diversity, Recombination, Adaptive evolution, Ecological differentiation

## Abstract

**Background:**

*Micrococcus luteus* is a group of actinobacteria that is widely used in biotechnology and is being thought as an emerging nosocomial pathogen. With one of the smallest genomes of free-living actinobacteria, it is found in a wide range of environments, but intraspecies genetic diversity and adaptation strategies to various environments remain unclear. Here, comparative genomics, phylogenomics, and genome-wide association studies were used to investigate the genomic diversity, evolutionary history, and the potential ecological differentiation of the species.

**Results:**

High-quality genomes of 66 *M. luteus* strains were downloaded from the NCBI GenBank database and core and pan-genome analysis revealed a considerable intraspecies heterogeneity. Phylogenomic analysis, gene content comparison, and average nucleotide identity calculation consistently indicated that the species has diverged into three well-differentiated clades. Population structure analysis further suggested the existence of an unknown ancestor or the fourth, yet unsampled, clade. Reconstruction of gene gain/loss events along the evolutionary history revealed both early events that contributed to the inter-clade divergence and recent events leading to the intra-clade diversity. We also found convincing evidence that recombination has played a key role in the evolutionary process of the species, with upto two-thirds of the core genes having been affected by recombination. Furthermore, distribution of mammal-associated strains (including pathogens) on the phylogenetic tree suggested that the last common ancestor had a free-living lifestyle, and a few recently diverged lineages have developed a mammal-associated lifestyle separately. Consistently, genome-wide association analysis revealed that mammal-associated strains from different lineages shared genes functionally relevant to the host-associated lifestyle, indicating a recent ecological adaption to the new host-associated habitats.

**Conclusions:**

These results revealed high intraspecies genomic diversity of *M. luteus* and highlighted that gene gain/loss events and extensive recombination events played key roles in the genome evolution. Our study also indicated that, as a free-living species, some lineages have recently developed or are developing a mammal-associated lifestyle. This study provides insights into the mechanisms that drive the genome evolution and adaption to various environments of a bacterial species.

**Supplementary Information:**

The online version contains supplementary material available at 10.1186/s12864-021-07432-5.

## Background

*Micrococcus luteus*, the type species of the genus *Micrococcus* (family *Micrococcaceae*, order *Micrococcales*), is a high GC Gram-positive coccus of the phylum *Actinobacteria* [1]. *M. luteus* is known as an opportunistic pathogen for nosocomial infections [2], and has been proved to be able to cause bacteremia, pneumonia, endocarditis, lymphoma, septic arthritis and many other diseases [3–5]. Besides as a pathogen, *M. luteus* is ubiquitously distributed in a variety of habitats, including soil, air, marine, plant and the human body [6], indicating that this species has been well adapted to various environments. It was also found that *M. luteus* can resuscitate and stimulate ‘viable but non-culturable (VBNC)’ or uncultured bacteria, by secreting a small protein called resuscitation-promoting factor (Rpf) [7, 8], and this feature has already been used in many biotechnological fields [9–11]. Therefore, as an emerging nosocomial pathogen and a strain of biotechnological interest, *M. luteus* has received increasing attention in recent years. It was speculated that *M. luteus* is primarily adapted to mammalian skin, and that its occasional presence elsewhere, such as water or soil, might possibly arise from contamination by skin flakes [12]. However, the adaptation strategy to various environments and the underlying genetic basis remains largely unknown.

Comparative and population genomics have emerged as valuable tools to delimit species features and to explore mechanisms of environmental adaptation or even speciation. With the ever-increasing whole-genome sequencing of closely related populations of microorganisms, combined with simulations and modelling, it has been widely accepted that microbial speciation is usually driven by natural selection for adaptation to distinct ecological niches [13, 14]. During this process, genomic variation caused by horizontal transfer (gene gain), gene loss, and duplication, plays a significant role [15, 16]. For example, a population genomics study of vibrios revealed a large number of gene gain and loss events in their evolutionary history, enabling vibrios to occupy various niches [17]. Similarly, it was also shown that, in streptomycetes, genomic fluctuation could ensure a quick and economical response to various lifestyles [18, 19]. The first sequenced genome of *M. luteus* revealed a circular chromosome with one of the smallest genomes of free-living actinobacteria and an abnormally high number of transposable elements [12]. However, the genomic diversity of *M. luteus* and the genomic events that contribute to the ecological adaptation need further study.

Now genome sequences of more than 70 *M. luteus* strains have been published and/or are available publicly [20–22], providing an opportunity to systematically study its physiology, ecology, and evolution at the genomic level. This study was aimed to investigate the intra-species genomic diversity of *M. luteus* and to uncover the evolutionary events that may contribute to the genomic diversity. Furthermore, this study was also intended to explore the relationships between the intra-species genomic diversity and the potential ecological differentiation within the species.

## Results

### Genomic features of *M. luteus*

**Fig. 1:**
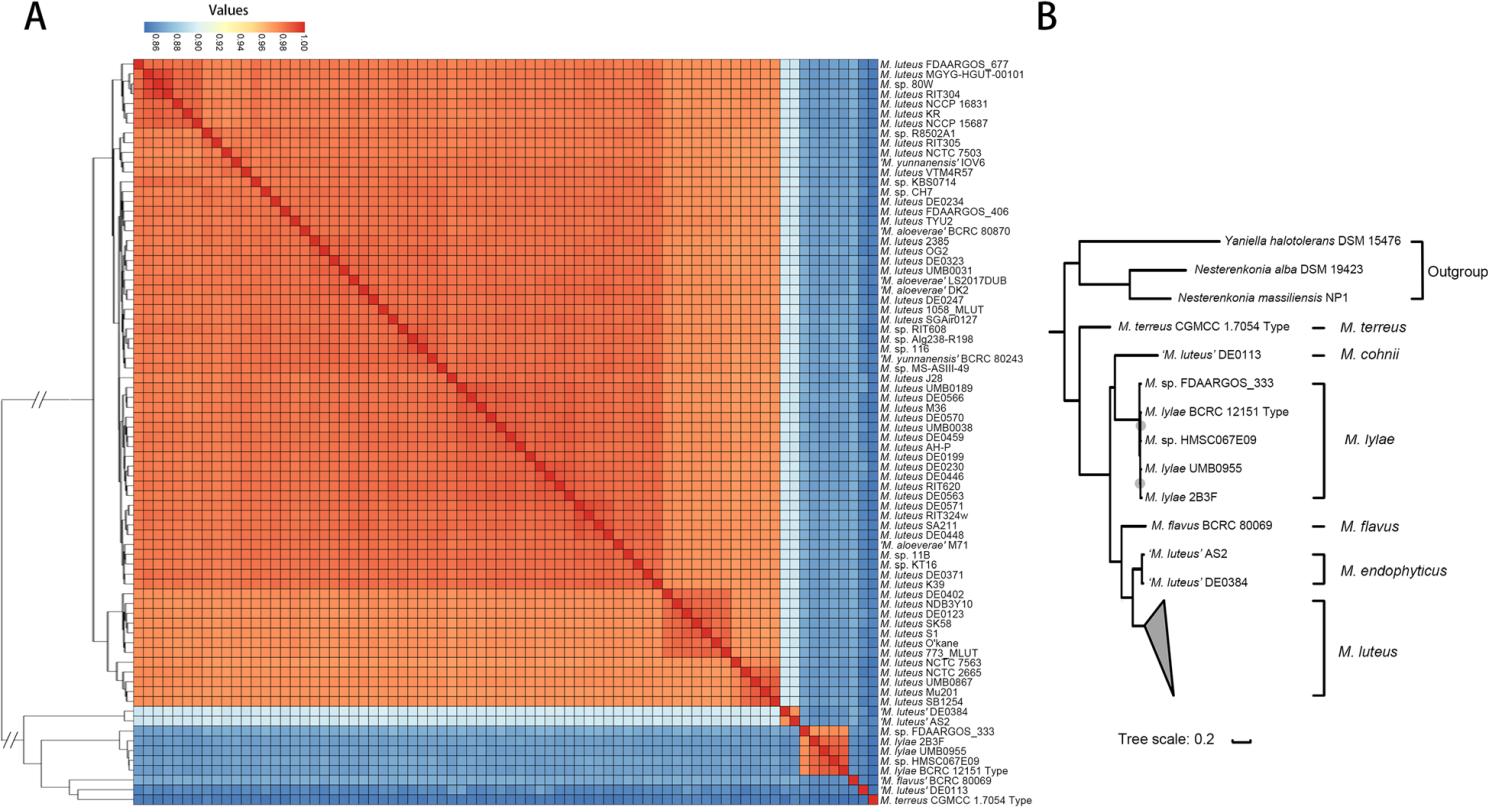
Pairwise genome-wide ANI values and maximum-likelihood phylogeny of the genus *Micrococcus*. **a** Heatmap of average nucleotide identities between *Micrococcus* strains used in this study. ANI values calculated by the pyani are color-coded according to the provided scale bar. **b** Maximum-likelihood phylogeny generated from concatenated 174 single-copy core genes with no evidence of recombination from PhiPack of the 76 *Micrococcus* strains, including three outgroup strains. Bootstrap values less than 90% are shown by gray dots at the nodes. The scale bar indicates 20% sequence divergence

All (106) *Micrococcus* genome sequences available at the NCBI (National Center for Biotechnology Information) assembly database (April 2020) were downloaded and subject to strict quality control to reduce potential bias in the subsequent analyses due to different assembly qualities (see Methods for details). As a result, 76 high-quality (completeness > 95% and contamination < 5%) and non-redundant *Micrococcus* genomes remained (Table S1). Whole-genome average nucleotide identity (ANI) analysis classified all these genomes into six clades (species) based on an ANI cutoff of 95% (Fig. 1a), three of which (including 72 strains) corresponded to three published species with inner ANI > 97.4%: *M. luteus* (*n* = 66), *M. lylae* (*n* = 5), and *M. terreus* (*n* = 1), respectively. As the other three clades did not contain type strain genomes, the remaining four strains (*‘Micrococcus luteus’* AS2, *‘Micrococcus luteus’* DE0384, *‘Micrococcus flavus’* BCRC 80069, and *‘Micrococcus luteus’* DE0113) in these three clades were then assigned to known species by comparing their near full-length 16S rRNA gene sequences to those of type strains. Strains AS2 and DE0384 were reclassified into *M. endophyticus* (type strain: YIM 56238^T^, identity > 99.5%, 1432 nt) [23], strain DE0113 was reclassified into *M. cohnii* (type strain: WS4601^T^, identity = 100%, 1109 nt) [24], and strain BCRC 80069 was reclassified into *M. flavus* (type strain: LW4^T^, identity = 99.9%, 1402 nt) [25]. Further phylogenomic analysis supported the species classification (Fig. 1b).

Our dataset contained genomes for 66 *M. luteus* strains. These strains were from diverse habitats globally, including marine, soil, food, plant- and human-associated, and other common sources (Table S1). Compared to high intra-species ANI values (> 96.8%), the ANI values with other species were much lower (< 89.4%), suggesting a clear inter-species genomic difference. The *M. luteus* genome size ranged from 2.41 Mb (strain OG2) to 2.74 Mb (strain DE0230) (Figure S1A), with an average genome size (2.52 Mb, *n* = 66) smaller than *M. lylae* (2.68 Mb, *n* = 5; *p* = 0.001, Wilcoxon test), *M. endophyticus* (2.78 Mb, *n* = 2; *p* = 0.02, Wilcoxon test), and *M. terreus* (3.09 Mb, *n* = 1), but larger than *M. cohnii* (2.24 Mb, *n* = 1). In addition, the GC content of *M. luteus* (mean 72.9%, *n* = 66; ranging from 72.3 to 73.3%) was higher than *M. lylae* (mean 71.3%, *n* = 5; *p* = 0.0002, Wilcoxon test), *M. cohnii* (70.8%, *n* = 1) and *M. terreus* (68.9%, *n* = 1), but lower than *M. endophyticus* (mean 73.4%, *n* = 2; *p* = 0.02, Wilcoxon test) and *M. flavus* (73.5%, *n* = 1) (Figure S1B). Taken together, the differences in ANI values, genome size, and GC content, suggested an apparent divergence between *M. luteus* and the other species of *Micrococcus*.

The *M. luteus* genomes each had 2148 (strain OG2) to 2501 (strain SGAir0127) coding sequences (CDSs) (mean 2287). On average, 94.6% of them could be annotated and functionally categorized using the eggNOG database [26]. Each genome contained 159 to 315 identifiable insertion sequences (ISs), accounting for 7.22 to 13.4% of the genome (mean 8.51%). Each genome also harbored 4 to 15 genomic islands (GIs; mean 8.3) that were 3.1 to 96.2 kbp in size, which together accounted for an average of 6.6% of the whole genome (ranging from 2.3 to 11.4%). These results suggested that strains of *M. luteus* might have undergone frequent genomic exchanges [27]. In addition, 57 strains (86.4%) encoded Rpf, a secreted protein with an N-terminal transglycosylase-like domain (PF06737) and a C-terminal LysM domain (PF01476), which could stimulate the growth and resuscitation of dormant bacteria [7, 8]. This dormancy-resuscitation mechanism may help *M. luteus* to survive over extended periods when conditions are not conducive for growth, and to rapidly respond to environmental changes.

### Potential virulence factors and antibiotic resistance genes of *M. luteus*

All *M. luteus* genomes were locally compared against the Virulence Factors Database (VFDB) [28] to detect virulence genes (Figure S2). We found 30 different putative virulence factors (VFs), twelve of which (40%) were related to GIs. Each genome contained 19 (strains RIT608 and 1058_MLUT) to 31 (strain SK58) VF genes (mean 22). Twelve VFs were shared by all strains, of which three (*clpC*, *clpP*, *katA*) were involved in stress response, three (*ideR*, *phoP*, *relA*) in regulation, two (*lirB*, *CBU_1566*) in secretion systems, and the remaining four (*htpB*, *gnd*, *bauE*, *icl*) in adherence, immune evasion, iron uptake and metabolic adaptation, respectively. These core VFs might play important roles in the pathogenicity of *M. luteus*. Copy number variation of the VF genes was also found. For example, genes *ureABG* were enriched (three to six copies) in strain SK58 (isolated from human skin). These genes are involved in bacteria urea hydrolysis and have been reported to be crucial to pathogenic bacteria virulence and defense against host immunity [29, 30]. Genes *msrA/B* (*pilB*) were expanded (three copies) in five strains and their gene products can promote the successful infection of humans and also respond to adverse conditions [31, 32]. The expansion of VF genes may promote the pathogenicity of the harboring strains.

We also detected the presence of antibiotic resistance genes (ARGs) in *M. luteus*. A total of 22 distinctive putative ARGs were identified, half of which were related to GIs (Figure S3). Overall, three ARGs were identified in all strains, including genes associated with macrolide- and penam-resistance (*mtrA*, with two copies), fosfomycin-resistance (*murA*) and rifamycin-resistance (*rbpA*). Additionally, an aminoglycoside phosphotransferase encoded by *strA*, which confers aminoglycoside-resistance, was found in 59.1% (*n* = 39) of the strains. The remaining genes (*n* = 18, 81.8%) exhibited sporadic distribution patterns (including eight strain-specific genes). These genes included 13 antibiotic efflux pump-encoding genes, three antibiotic inactivation enzymes encoding genes (*aac (3)-IIb*, *blaCTX-M-141*, *blmS*), and two genes for antibiotic target alteration (*rmtF*) and protection (*msrE*). Different ARG repertoire suggested that different strains may have different antibiotic resistance.

### Core- and pan-genome of *M. luteus*

**Fig. 2:**
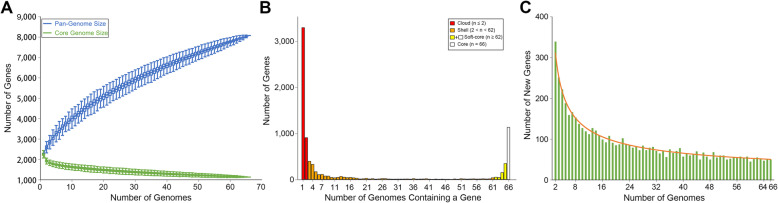
The pan-genome, core genome, and accessory genome profiles of *M. luteus*. **a** The sizes of pan- and core-genomes in relation to numbers of genomes added into the gene pool. **b** Distribution of genes across strains. Cloud, shell, and (soft-) core pangenome components were derived according to GET_HOMOLOGUES. **c** Number of new genes for each additional genome analyzed

To explore the entire genomic repertoire of the *M. luteus* population, estimates of the cloud (genes present in only one or two genomes), shell (genes present in 3–61 genomes), softcore (genes present in at least 62 genomes) and core (genes present in all 66 genomes) genomes were generated using GET_HOMOLOGUES [33]. The 66 *M. luteus* strains had a pan-genome of 8077 genes and a core genome of 1134 (14.0%) genes, including 991 single-copy core genes (Fig. 2). The core genome only represented 45.3 to 52.8% of the gene content of each strain, illustrating a relatively high degree of genomic diversity. This core gene ratio was much lower than that of the actinobacterial species *Streptomyces albidoflavus* (65.3 to 73.0%, recalculated with the same clustering algorithm and parameters, intraspecies ANI > 98.1%) [18]. Furthermore, the cloud genes contained more than half of the pan-genome (4210 genes, 52.1%; 3.36% for each strain, on average), of which 3301 were strain-specific genes (singletons, 40.9%; 2.14% for each strain, on average), indicating an exceptionally flexible genome of *M. luteus*. Correspondingly, the pan-genome for *M. luteus* still increased with approximately 50 new genes after addition of a 66th genome (Fig. 2c). Analysis of the pan-genome curve using a power-law regression model confirmed that the pan-genome was open (*B*_pan_ = 0.5), as the curve did not approach a constant as more genomes were selected.

We next performed a Clusters of Orthologous Groups (COG) functional classification for each orthologous group (OG) to define possible differences in the functions encoded by the soft-core, cloud and shell genomes of *M. luteus* (Figure S4). As a result, the soft-core genome had a higher proportion of genes classified in COG categories C (energy production and conversion), E (amino acid transport and metabolism), F (nucleotide transport and metabolism), H (coenzyme transport and metabolism) and J (translation, ribosomal structure and biogenesis) (*p* < 0.01, Fisher’s exact test), all associated with basic biological functions. The cloud and shell genes were biased toward COG categories L (replication, recombination and repair) and V (defense mechanisms) (*p* < 0.01, Fisher’s exact test), which may contribute to the intraspecies heterogeneity of the species, as these genes have been proved to play important roles in the acquisition of foreign DNA [34].

### Phylogenomic relationship and population structure of *M. luteus*

**Fig. 3:**
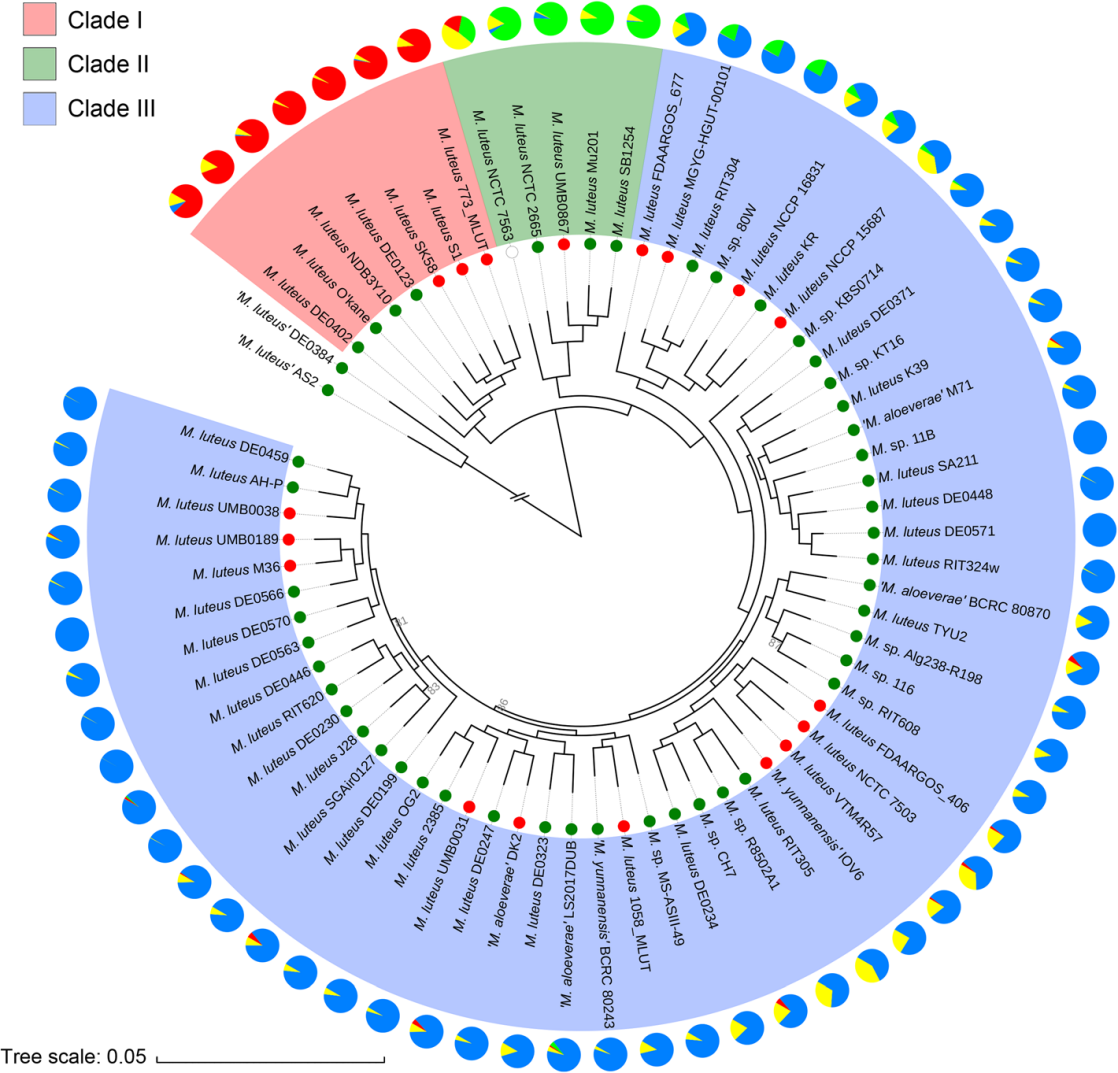
Phylogenetic tree and the population structure of the *M. luteus* strains. Maximum-likelihood phylogeny was generated from concatenated 922 single-copy core genes of 68 strains, including the two outgroup strains. Three major clades were presented by different background colors. All nodes have at least 90% bootstrap support, except where indicated. Different habitats are represented by inner circles in different colors (red, HA; green, NHA; blank, uncertain). The output from Structure analysis was indicated in the pie charts with colors indicating the proportion of ancestry estimated from each of four hypothetical ancestral populations (red, POP1; green, POP2; blue, POP3; yellow, POP4). The scale bar indicates 5% sequence divergence

To uncover the evolutionary process that led to the genomic diversity, we constructed a highly robust phylogenomic maximum-likelihood (ML) tree of the 66 *M. luteus* strains based on concatenated 922 single-copy core genes, using the two *M. endophyticus* strains (‘*Micrococcus luteus*’ AS2 and ‘*Micrococcus luteus*’ DE0384) as outgroups (Fig. 3). Based on the phylogenomic tree, *M. luteus* could be divided into three basal clades: Clade I, II, and III. Further analyses revealed clear inter-clade genomic boundaries. Firstly, the ANI heatmap and clustering results (Fig. 1a) suggested that the species could be divided into three groups, with high intra-group ANI and relatively low inter-group ANI. Comparison of this clustering result with the phylogenomic tree indicated that the three clades corresponded nicely with the three ANI groups, indicating the existence of inter-clade ANI boundaries. Secondly, in order to investigate whether there were gene content differences between the three clades, a hierarchical clustering tree based on the content of dispensable genes was constructed (Figure S5). This tree clearly showed three clades, which were the same as those in the core genome tree, indicating the existence of inter-clade gene content boundaries. These results suggested that the separation between the three clades has emerged, both in the core and accessory genes.

The intraspecies difference was also studied using methods which are widely used in population structure analyses. By using Fastbaps [35], the entire *M. luteus* population could be partitioned into three subpopulations at Level 2 (Figure S6). These three subpopulations corresponded to the three major clades on the phylogenomic tree. At Level 3 or higher, one additional subpopulation emerged, which corresponded to only strain NCTC 7563 in Clade II. Furthermore, an admixture model implemented in STRUCTURE [36] software was applied, which showed maximal posterior probability at K = 4, indicating the existence of four ancestral subpopulations (Fig. 3 and Figure S6). Among them, three ancestral subpopulations (POP1, POP2, and POP3) were mainly represented by the three major clades. It was noted that, the fourth ancestral subpopulation (POP4, yellow) existed in high proportions in all the three clades, with the highest proportion in the fourth subpopulation uncovered by the above Fastbaps analysis (i.e., strain NCTC 7563 in Clade II). However, since POP4 accounted for only 47.5% of the genome of NCTC 7563, it is unclear whether POP4 can be represented by the branch of strain NCTC 7563. It is possible that POP4 corresponded to an unsampled clade.

### Gain and loss of genes during the evolution of *M. luteus*

To unravel the evolutionary events that contributed to the intraspecies gene content differences, gene contents of all ancestral nodes on the phylogenomic tree were predicted and the numbers of gene gain and loss events that have occurred on all branches were calculated using a parsimony method (Figure S7). As a result, the last common ancestor of *M. luteus* was inferred to possess 2028 gene families. The gene family numbers had only slightly increased during evolution, as the gene gain events were a bit more numerous than the gene loss events in most stages during the evolutionary process. Also, a relatively high number of gene content variances occurred at the divergence of the three major clades (77, 64 and 55 for Clades I to III, respectively), consistent with inter-clade gene content differences mentioned above (Figure S5). Interestingly, 88.3% of total gene gain events and 82.8% of total gene loss events occurred at terminal nodes, suggesting that the pan-genome diversity of *M. luteus* is largely due to frequent but recent strain-specific gene gain/loss events.

### High level of homologous recombination in *M. luteus* species

**Fig. 4:**
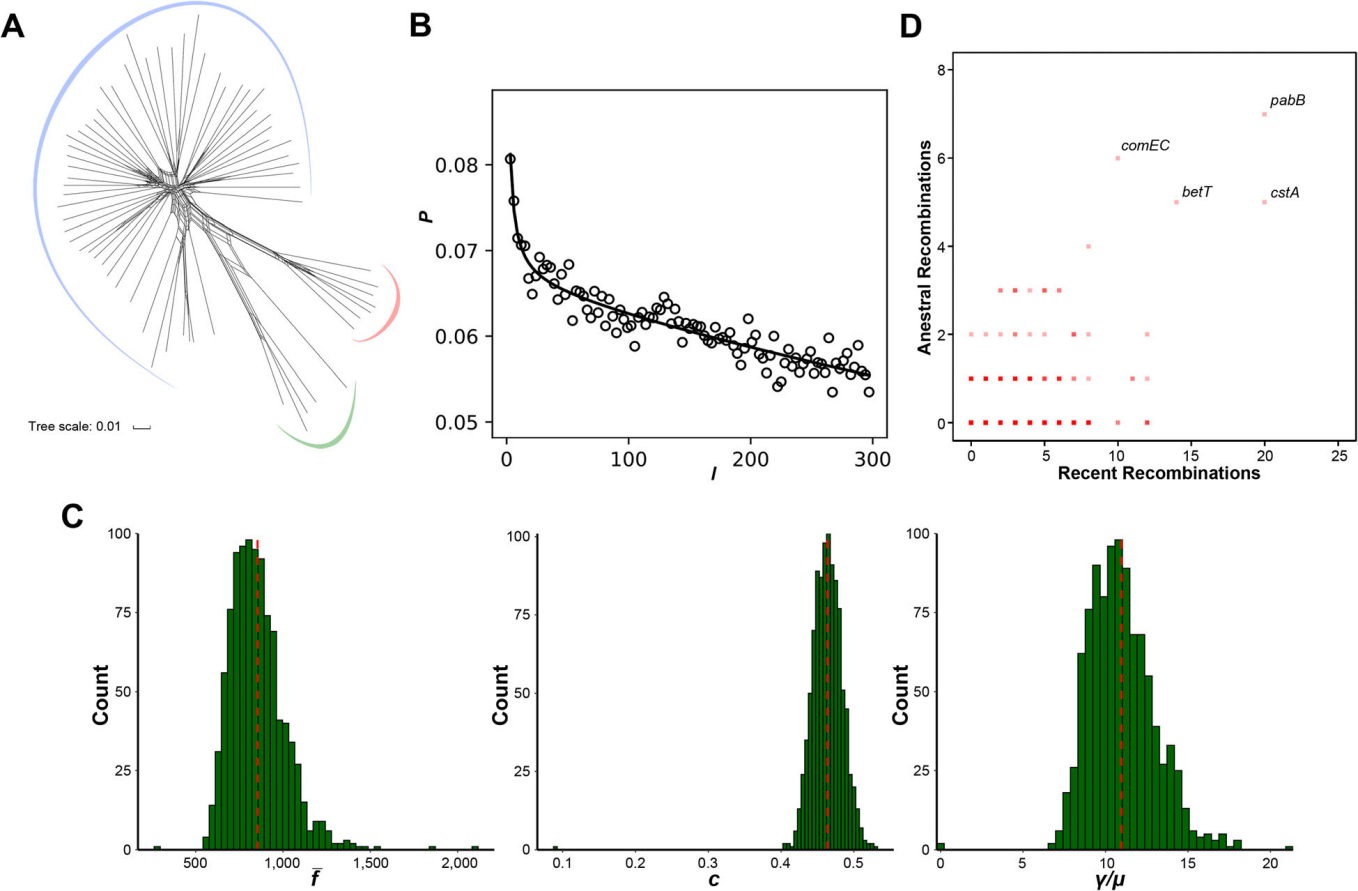
Recombination in *M. luteus*. **a** Phylogenetic network inferred with the concatenation of single-copy core genes. Lines between splits show where recombination has occurred. Three major clades are presented by different colored arcs. The scale bar indicates 1% sequence divergence. **b** Correlation profile (circles) calculated by mcorr. Model fit is shown as a solid line. **c** Distributions of the three recombination parameters for all pairs of genomes. Red vertical lines indicate the means calculated with 1000 bootstrapped replicates. **d** Core genes that have undergone recent and ancestral recombination. Horizontal and vertical axes show the estimated number of recent and ancestral recombinations, respectively. Names of some of the most frequently recombined genes are shown

Recombination, especially homologous recombination, is one of the main forces shaping bacterial evolution [37, 38]. By recombination, bacteria can receive DNA fragments from both the same and other species and integrate them into their chromosomes. Here, a series of analyses were used to evaluate the extent of homologous recombination in the core genome of *M. luteus*. Firstly, a NeighborNet network [39] based on the concatenated single-copy core genes showed a reticulate structure, indicating a high level of non-vertical inheritance in phylogenies (Fig. 4a). Meanwhile, the pairwise homoplasy index (PHI) [40] statistic provided highly significant evidence for recombination within *M. luteus* species (*p* = 0.00).

We also used mcorr [41] to calculate the probability that a pair of genomes differs at one locus conditional on having differences at the other locus. The resulting correlation profile exhibited a monotonic decay (Fig. 4b), indicating the presence of recombination. Similar decaying correlation profiles have also been shown in other recombining bacteria, such as *Helicobacter pylori*, *Pseudomonas aeruginosa*, *Salmonella enterica* and *Cronobacter sakazakii* [41–43]. Meanwhile, mcorr showed the mean fragment size (f̅) of a recombination event in *M. luteus* species was 874.45 bp; the recombination coverage (c), which indicates the proportion of sites in the genome whose diversity has come from recombination events since its last common ancestor, was 0.46; and the ratio γ/μ, the relative rate of recombination to mutation, was 10.87 (Fig. 4c). All these recombination parameters above further confirmed a high recombination rate within *M. luteus*. A summary of the parameters of *M. luteus* and other typical species reported was shown in Table 1.

**Table 1:** Recombination parameters of different species inferred by mcorr

Species	f̅	c	γ/μ	References
*Acinetobacter baumannii*	860	0.40	1.30	[41]
*Campylobacter jejuni*	1000	0.32	3.40	[41]
*Cronobacter sakazakii*	816	0.53	1.61	[43]
*Klebsiella pneumoniae*	5800	0.27	4.20	[41]
*Micrococcus luteus*	874	0.46	10.87	This study
*Micrococcus luteus* (NHA only)	903	0.45	10.41	This study
*Micrococcus luteus* (HA only)	849	0.49	10.93	This study
*Mycobacterium abscessus*	1200	0.21	13.00	[41]
*Pseudomonas aeruginosa*	590	0.52	11.00	[41]
*Salmonella enterica*	–	0.46	6.16	[42]
*Staphylococcus aureus*	550	0.36	1.00	[41]
*Staphylococcus pseudintermedius*	407	0.24	3.97	[44]
*Yersinia pestis*	530	0.03	3.00	[41]

NHA, non-host-associated

HA, host-associated

-, information unavailable

We next sought to identify the frequently recombining genes. Among the 991 single-copy core genes, 628 genes (63.37%) showed significant evidence of genetic recombination, with *P*-values (computed from 1000 permutations) lower than 0.05 in at least two of the three methods implemented in PhiPack [40]. This result was further confirmed by fastGEAR [45], by which a total of 708 genes (71.44%) were detected to have been affected by recent or ancestral recombination events (Fig. 4d). The most frequently recombining genes included *pabB*, *cstA*, *betT*, and *comEC*: *pabB* encodes an enzyme that catalyzes the two-step biosynthesis of anthranilate and has experienced trans-kingdom gene fusions [46]; *cstA* is involved in peptide utilization during carbon starvation [47]; *betT* encodes a choline-glycine betaine transporter, which could help to overcome osmotic stress by the accumulation of compatible solutes [48, 49]; and *comEC* encodes a transformation protein and has been shown to be absolutely required for DNA uptake and recognition [50].

### Habitat-associated accessory OGs underlying putative differential adaptation

*M. luteus* is widely distributed in various environments and a few strains were isolated as human pathogens or from the mammalian skin or other tissues. In order to investigate whether the genomic differences between clades are relevant to the habitat, the isolation resources of all strains were mapped to the phylogenomic tree (Fig. 3 and Figure S6). Strains whose isolation sources were known could be classified into two groups: host-associated (HA), including 18 strains isolated from mammal hosts, and non-host-associated (NHA), including 47 strains isolated from other sources or free living. HA strains were not clustered on the tree but rather widely distributed in all clades, indicating the inter-clade genetic difference was irrelevant to the habitat transition. It was noted that HA strains tended to locate on the lately diverged branches, and that strains on the early diverged branches as well as the closest characterized relatives of *M. luteus* (*M. endophyticus* and *M. flavus*) were all isolated from NHA habitats. These results suggested that the last common ancestor of *M. luteus* was more likely to be genuine dwellers in NHA niches, and a few descendants transited to a mammal-associated lifestyle recently.

**Fig. 5:**
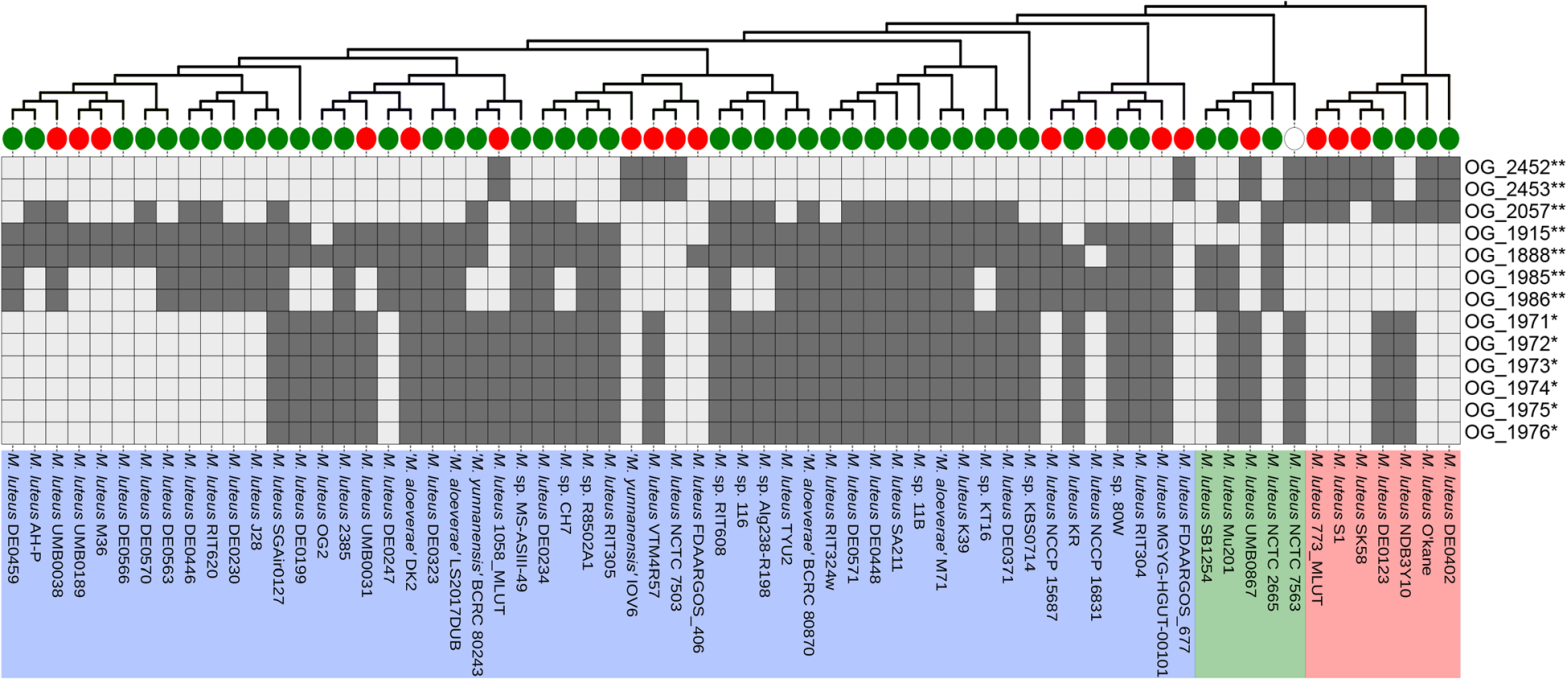
Distribution of the habitat-associated OGs. Details of these genes are shown in Table S2. Heatmaps represent the distribution of 13 accessory OGs that significantly associated with HA and NHA habitats. Dark and light gray boxes indicate presence and absence, respectively. The tree topology is derived from Fig. 3, and the typical clades are marked with different colors. Strains from different habitats are represented by circles in different colors (red, HA; green, NHA; blank, uncertain). Asterisks indicate the naive *P*-value for the null hypothesis that the presence/absence of this OG is unrelated to the habitat (*, *P* < 0.05; **, *P* < 0.01)

**Fig. 6:**
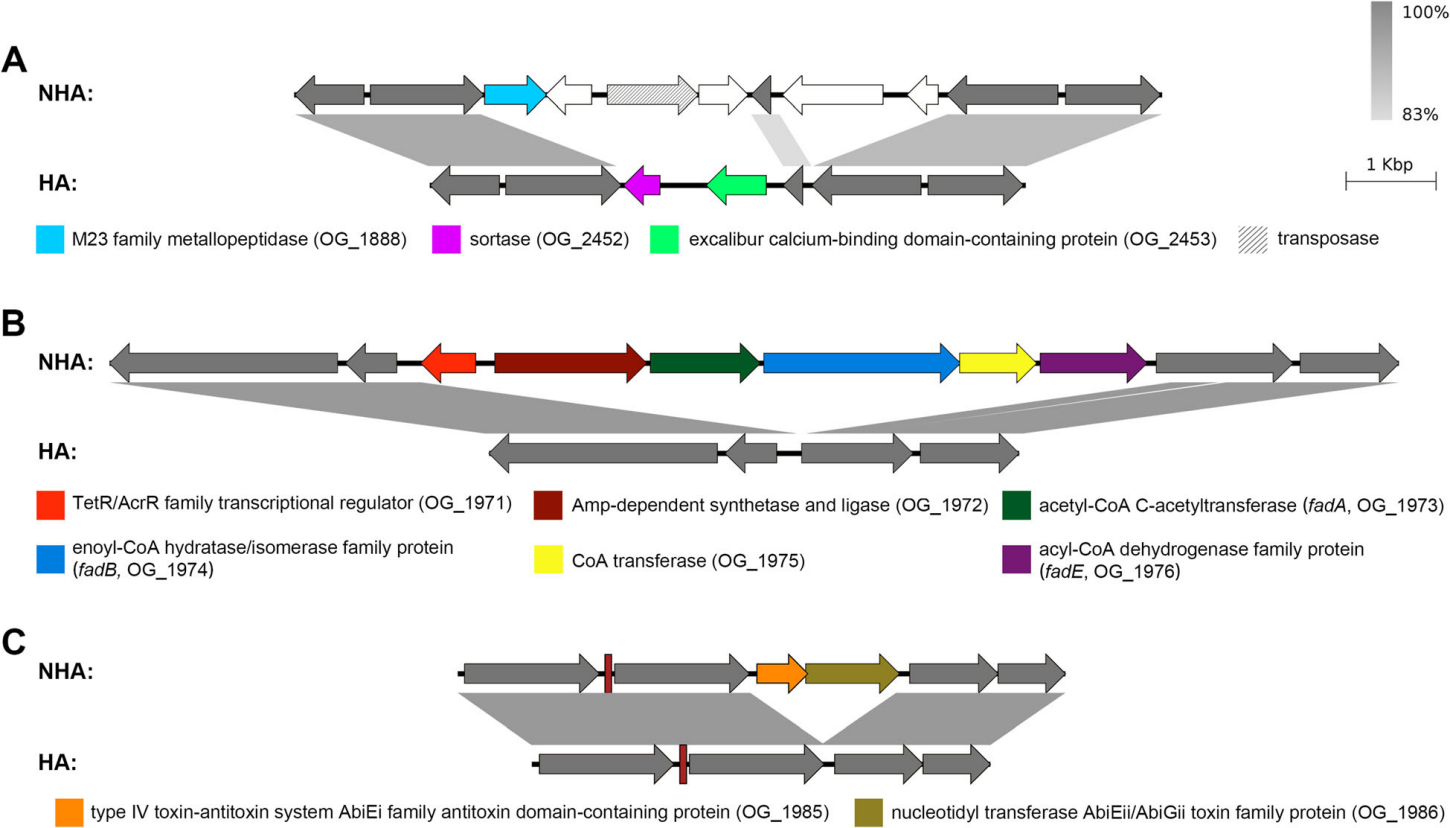
Genetic organizations of the regions containing habitat-associated genes. Details of these genes are shown in Table S2. The figure was obtained by EasyFig, where gray-scale bars represent regions of shared similarity according to BLASTn. Colored arrows indicate habitat-associated genes. Grey arrows indicate flanking and unrelated genes. **a** The versatile genomic region with both HA and NHA genes. Slash indicates transposase gene. Blank arrows indicate habitat unassociated genes. **b** The gene cluster for degradation and recycling of fatty acids via β-oxidation. **c** The gene cluster for bacterial abortive infection system. Slim rectangle indicates tRNA gene

Lifestyle changes are drastic events followed by gene gain and loss. We sought to study whether strains from different habitats were associated with different sets of genes. A pan-genome-wide association study (pan-GWAS) revealed 101 accessory genes that were present in at least half of the strains from one habitat but no more than half from the other. Among the 13 genes whose associations were statistically significant (Fig. 5 and Table S2, Benjamini-Hochberg *P*-value < 0.1 and Empirical *P*-value < 0.05), eleven were enriched in NHA strains and two were enriched in HA strains. It was noted that the two HA genes (OG_2452 and OG_2453) and one of the NHA genes (OG_1888) were located at the same genomic region (Fig. 6a). Genes of OG_2452 and OG_2453 encoded a sortase and an excalibur calcium-binding domain-containing protein, respectively. Sortases are enzymes responsible for covalent anchoring of specific proteins to the peptidoglycan of the cell wall of Gram-positive bacteria, performing critical biological functions that are required for the colonization and invasion of host tissues [51, 52]. Sortases have also been considered as important virulence factors, as they play key roles in the infection process [53]. The NHA gene OG_1888 encoded a protein that was a member of COG0739 of membrane proteins related to metallo-endopeptidases. However, the function of OG_1888 in the adaption to the NHA habitats is unknown. Considering the conserved flanking genes and the presence of a transposase gene inside the region, it is possible that the integration of this region was a result of horizontal gene transfer (HGT). Actually, the NHA gene OG_1888 was located within a putative GI, further supporting the hypothesis. Additionally, the OG_2452-OG_2453 gene cluster existed in HA strains located on different clades, indicating that different HA lineages obtained this gene cluster separately and that there may be HGT between different HA lineages.

Most NHA genes were clustered within two regions. One such region contained six NHA genes (OG_1971 to OG_1976) (Fig. 6b). In this region, three genes, *fadA*, *fadB*, and *fadE*, responsible for the degradation and recycling of fatty acids via β-oxidation, were identified. Fatty acids are essential components of membranes and are important sources of metabolic energy in all organisms [54]. The other region contained genes encoding a type IV toxin-antitoxin ‘innate immunity’ bacterial abortive infection (Abi) system, consisted of a bicistronic operon encoding an AbiEi antitoxin and an AbiEii toxin (OG_1985 and OG_1986, Fig. 6c). The Abi system, abbreviated for the phage abortive infection system, provides a post-infection resistance mechanism that could block phage multiplication and result in the death of the infected bacterial cell upon phage infection [55–57]. Beside these two genomic regions, there were also two genes (OG_1915 and OG_2057) showing a significant association with NHA habitat. One of these (OG_2057) encoded a glyoxalase (COG0346/PF18029), which has been reported to play a role in the detoxification of methylglyoxal in bacterial cell metabolism when suffering environmental stresses [58]. Thus, these NHA genes probably enhanced the adaptation to complex environments.

## Discussion

The ability of *M. luteus* to survive and reproduce in a wide range of ecological niches and to cause disease not only is practically significant, but also provides an ideal opportunity for inferring the population genetics and evolution of environmentally transmitted pathogens. In fact, *M. luteus* was once considered to be a non-pathogenic saprophyte or pure contaminant from skin and mucous membranes, but it is now proved to cause severe infections in immunocompromised populations [3–5]. In this study, a genomic investigation on VF and ARG profiles of *M. luteus* was performed. It was noted that about half the ARGs and VFs were related to GIs, suggesting that HGT might be an important driving force for antibiotic resistance and pathogenicity acquisition in *M. luteus*. In addition, most of the previously reported cases due to *M. luteus* could be successfully treated with a combination of vancomycin and rifampin [59], but there was also a report that a treatment regimen consisting of vancomycin, gentamicin, and rifampicin for 4 weeks was not successful in the case of native aortic valve endocarditis secondary to *M. luteus* [60], suggesting the antibiotic resistance mechanism of *M. luteus* is still very complicated. Consistently, our analyses showed that all of the total 66 genomes contained the *rbpA* gene, the product of which has been reported to confer basal levels of rifampicin resistance on *Streptomyces coelicolor* [61]. Therefore, the VF and ARG profiles may provide guidance for the future treatment of *M. luteus* infections. However, these results may be biased due to the incomplete genome sequences for most strains, though only high-quality genome sequences (completeness > 95% and contamination < 5%) were included in our dataset.

The present study also constitutes a comprehensive comparative genomic characterization of *M. luteus*. We show that *M. luteus* is characterized by high genomic diversity with large open pan-genome and high number of accessory and unique genes. This pan-genome diversity is considered an indicator of high HGT rates, especially for strains living in multiple niches [62, 63], suggesting that *M. luteus* had a high capacity to absorb and integrate external genetic elements from environments. This is further supported by the detection of extensive gene gain/loss events that have occurred along the evolutionary history. All the above results showed a considerable intraspecies heterogeneity of *M. luteus*, which has been preliminarily proposed based on macro-restriction analysis using pulsed-field gel electrophoresis [64]. This high genome variability may contribute to the functional diversity of *M. luteus* strains thriving in various habitats.

Phylogenomic analysis indicated that *M. luteus* has diverged into three well-differentiated clades, while population structure analysis revealed four admixed ancestral subpopulations, with three of them corresponding to the three clades, respectively, and the fourth (represented by strain NCTC 7563) corresponding to an unknown ancestor. Besides, since isolation source information for strain NCTC 7563 was missing in the current dataset, other information and more sampling are needed to clarify what the predicted POP4 really is. In summary, while the population structure analysis supported the inter-clade differences, it suggested the existence of an unknown ancestor that contributed to the diversity of the species or the existence of the fourth, yet unsampled, clade. Furthermore, consistent with the high proportion of admixture inferred from population assignment and also the numerous insertion sequences and transposases detected in the genomes, our study provides compelling evidence for high-level homologous recombination within *M. luteus*. This will probably allow frequent gene exchange between *M. luteus* strains and with other organisms. Recombination has been proved as an important driver of the evolution of most prokaryotes, and acquisition of novel alleles of existing genes will also accelerate ecological adaptation of bacterial populations [65, 66].

It has been shown that ecological niches affect the evolution of bacteria [67, 68]. Since the transition between HA and NHA lifestyles is a dramatic ecological change, adaptive evolutionary processes will occur [69]. Based on the distribution of HA and NHA *M. luteus* strains on the phylogenetic tree, we proposed that the last common ancestor of *M. luteus* was more likely to be genuine dwellers in NHA niches. This proposition is different from an early speculation that *M. luteus* was primarily adapted to (mammalian) skin, and its presence in water or soil might come from contamination by skin flakes [12]. By using pan-GWAS, we identified a number of genes that presumably contribute to adaptation of *M. luteus* to different lifestyles, although the clustering patterns of these genes do not exactly follow habitat. For example, the sortase probably enables HA strains better colonization on mammalian hosts [52], and the Abi system may act as stress-response elements that helps NHA strains survive unfavorable growth conditions [57, 70]. Nevertheless, much of the inferred habitat-associated genes remain uncharted and/or await experimental verification. These genes suggested that the nascent ecological differentiation has already been initiated and pathogenic strains and lineages in this species are merging. However, it is unclear whether the differentiation has been completed. It has been proposed that microbial speciation is usually driven by natural selection for adaptation to distinct ecological niches, and the distinctness is further maintained by barriers to gene flow [71]. This process is not necessarily continuous and complete, and may be terminated at any time [14]. In fact, we did not detect any clues to the existence of recombination barriers between habitats, as there was no obvious difference in recombination parameters between datasets of HA, NHA, and all strains (Table 1). Here, using *M. luteus*, we have shown another case to depict the process of bacterial ecological differentiation, whether or not it proceeds to completion. During this process, niche-specifying (adaptive) variation has already emerged. However, the separation process seems to be suppressed probably because of the high-level recombination and the strong diffusibility between niches.

## Conclusions

We performed comprehensive comparative genomic analyses of 66 *Micrococcus luteus* genomes. Our study revealed high intraspecies genome diversity and extensive gene gain/loss events that contributed to the genome diversity, and showed that extensive recombination played key roles in the evolution of the species. Our results suggested that the last common ancestor of *Micrococcus luteus* had a free-living lifestyle and a few lineages have recently developed or are developing a mammal-associated lifestyle independently. This recent ecological differentiation appeared to be a process that was not related to the early phylogenetic separation of different clades of the species. This study highlighted the complicated evolution history of an emerging nosocomial pathogen *Micrococcus luteus* and provided new insights into mechanisms that drive the diversification of a species and adaptation to various environments.

## Methods

### Genome data set

All genomes of the members of the genus *Micrococcus* (*n* = 106, April 2020) that derived from different projects and habitats were downloaded from the NCBI genomes FTP site (https://www.ncbi.nlm.nih.gov/genomes). In order to ensure the reliability of the results, we used CheckM [72] to assess isolate genome completeness and contamination. Only genomes that were at least 95% complete and had no more than 5% contamination were used. In addition, we computed whole genome ANI for each pair of genomes by pyani (https://github.com/widdowquinn/pyani) in ANIm mode. To avoid the statistical deviation caused by highly similar strains or even multiple sequencing of the same strain (e.g., strain NCTC 2665), we considered genome pair redundant when ANI was higher than 99.9%. In such cases, one genome with the most completeness and the largest N50 was selected, and the others were marked as “redundant” and were filtered out. Through this, a total of 76 high-quality and non-redundant *Micrococcus* genomes were retained. The information of isolation source of each strain was obtained from the NCBI BioSample and BioProject databases and from the literature. Similarity searches for the 16S rRNA gene sequences were performed by the EzBioCloud server [73].

### Genome annotation and determination of OGs

To maintain consistency in gene annotations and to reduce the system errors caused by different programs or parameters, all genomes were reannotated locally and analyzed using the same strategy. De novo gene predictions for CDSs were performed with PRODIGAL [74]. Function annotation and classification of proteins were performed by sequence comparison against the eggNOG database [26], using the eggNOG-Mapper [75] with the DIAMOND method [76]. If a gene was assigned to more than one COG category, each COG category was calculated separately. Pfam domains were further identified by hmmscan in HMMER [77] against the PFAM-A database [78] with E-value ≤1e-05. Insertion sequences and transposases were identified by BLASTp against the ISFinder database [79] with manual inspection of search hits (E-value ≤1e-05). GIs were predicted using IslandPATH-DIMOB [80]. Potential ARGs and putative VFs encoded in *M. luteus* genomes were identified through BLASTp searches of the Comprehensive Antibiotic Resistance Database (CARD) [81] and the VFDB [28], respectively, with E-values ≤1e-05 and *Ha*-value > 0.42 [82]. Heatmaps were created by the pheatmap package in R (http://cran.r-project.org/web/packages/pheatmap/index.html).

All proteins were clustered using GET_HOMOLOGUES [33] to identify OGs with the OrthoMCL clustering algorithm. A relatively rigorous standard with 70% sequence identity and 70% coverage as minimum BLASTp homology cutoff was used. Cloud, shell, and (soft-) core pangenome components were derived according to GET_HOMOLOGUES. Pan-genome statistics were visualized using PanGP [83].

### Phylogenomics and population structure analyses

For phylogenomic analyses, only single-copy core genes were used. Codon-based alignments for each OGs were obtained by aligning the translated protein with MAFFT [84] and back-translating with PAL2NAL [85]. Poorly aligned regions were filtered by Gblocks [86], with default parameters except for option -t = c. The aligned sequences were concatenated as a single data set using homemade Python scripts. The approximately-maximum-likelihood phylogenetic tree was built using the generalized time-reversible model implemented in FastTree [87] and visualized using the Interactive Tree of Life [88]. In addition, we used R function pvclust [89] to perform a hierarchical cluster analysis, based on an absence/presence (0/1) matrix of dispensable genes according to GET_HOMOLOGUES results. To further elucidate the population structure of *M. luteus*, we used Fastbaps [35] with default parameters based on the core genome alignment. We also used the model-based Bayesian method implemented in Structure 2.3.4 [36], in which the admixture model was used with a varying K from 2 to 10, and the optimal value for K was obtained by STRUCTURE HARVESTER [90]. The pangenome matrix and the rooted species tree were used as inputs for COUNT [91] to calculate posterior probabilities for gain and loss of each OG across all nodes during the evolution of *M. luteus*.

### Estimation of genetic recombination

Four approaches were used to detect recombination in *M. luteus*. Based on the concatenated single-copy core genes, we firstly used SplitsTree [39] to construct a network with the NeighborNet algorithm, and also to calculate the pairwise homoplasy index [40]. Then, we ran fastGEAR [45] to detect recent and ancestral recombination events happening in each single-copy core gene with default parameters. In addition, PhiPack [40] was also used, by which potential recombination events were identified by having *P*-values lower than 0.05 in at least two of the three methods (PHI, Neighbor Similarity Score and Maximum Chi-Square) computed from 1000 permutations. Finally, we used mcorr [41] with default parameters to calculate correlation profiles and the recombination parameters, including the mean fragment size of a recombination event (f̅), the fraction of sample diversity derived from recombination (c), and the relative rate of recombination to mutation (γ/μ).

### Identification of the habitat-associated genes

Firstly, two 0/1 matrices were created: one based on the presence/absence of candidate habitat-associated genes (present in at least half of the strains of one habitat but no more than half of the other), and the other based on the habitat information of each strain, respectively. Then, a pan-GWAS using SCOARY [92] was carried out with 1000 permutation replicates to identify genes that significantly associated with HA or NHA lifestyle. Genes associated with different lifestyles were identified by odds ratio, and were considered as significant only when they attained a naive *P*-value less than 0.05, an empirical *P*-value less than 0.05, and a Benjamini-Hochberg corrected *P*-value less than 0.1. Pairwise comparisons of specific genomic regions within representative *M. luteus* strains were visualized using EasyFig [93].

## Supplementary Information

**Additional file 1: Table S1**. Genomic features and isolation sources of strains used in this study

**Additional file 2: Figure S1**. Variations in genome size and GC content between species in genus *Micrococcus*. **Figure S2**. Heatmap of the distributions of the putative VF genes in *M. luteus* genomes. **Figure S3**. Antibiotic resistance gene profiles of *M. luteus* strains. **Figure S4**. Comparison of the soft-core, shell and cloud genomes of *M. luteus* based on COG categories. **Figure S5**. Hierarchical cluster analysis based on the presence or absence of dispensable genes. **Figure S6**. Population structure of *M. luteus*. **Figure S7**. Ancestral genome content reconstruction using COUNT software

**Additional file 3: Table S2**. Accessory genes associated with HA or NHA habitats in *M. luteus*

## Data Availability

All data analyzed during this study are available through NCBI GenBank database, and are accessible through the accession numbers listed in Table S1.

## Abbreviations

Abi: Abortive infection
ANI: Average nucleotide identity
ARG: Antibiotic resistance gene
CARD: Comprehensive Antibiotic Resistance Database
CDS: Coding sequence
COG: Clusters of Orthologous Groups
GI: Genomic island
HA: Host-associated
HGT: Horizontal gene transfer
IS: Insertion sequence
ML: Maximum-likelihood
NCBI: National Center for Biotechnology Information
NHA: Non-host-associated
OG: Orthologous group
pan-GWAS: Pan-genome-wide association study
PHI: Pairwise homoplasy index
Rpf: Resuscitation-promoting factor
VBNC: Viable but non-culturable
VF: Virulence factor
VFDB: Virulence Factors Database
