# Supplementary material for: Comparative genomics reveals broad genetic diversity, extensive recombination and nascent ecological adaptation in Micrococcus luteus
Source: BMC Genomics. 2021 Feb 18;22:124. doi: 10.1186/s12864-021-07432-5 (PMC7890812; doi:10.1186/s12864-021-07432-5)
Supplement: Supplementary file 2 — Additional file 2: Figure S1. Variations in genome size and GC content between species in genus Micrococcus. Figure S2. Heatmap of the distributions of the putative VF genes in M. luteus genomes. Figure S3. Antibiotic resistance gene profiles of M. luteus strains. Figure S4. Comparison of the soft-core, shell and cloud genomes of M. luteus based on COG categories. Figure S5. Hierarchical cluster analysis based on the presence or absence of dispensable genes. Figure S6. Population structure of M. luteus. Figure S7. Ancestral genome content reconstruction using COUNT software [file 12864_2021_7432_MOESM2_ESM.pdf]

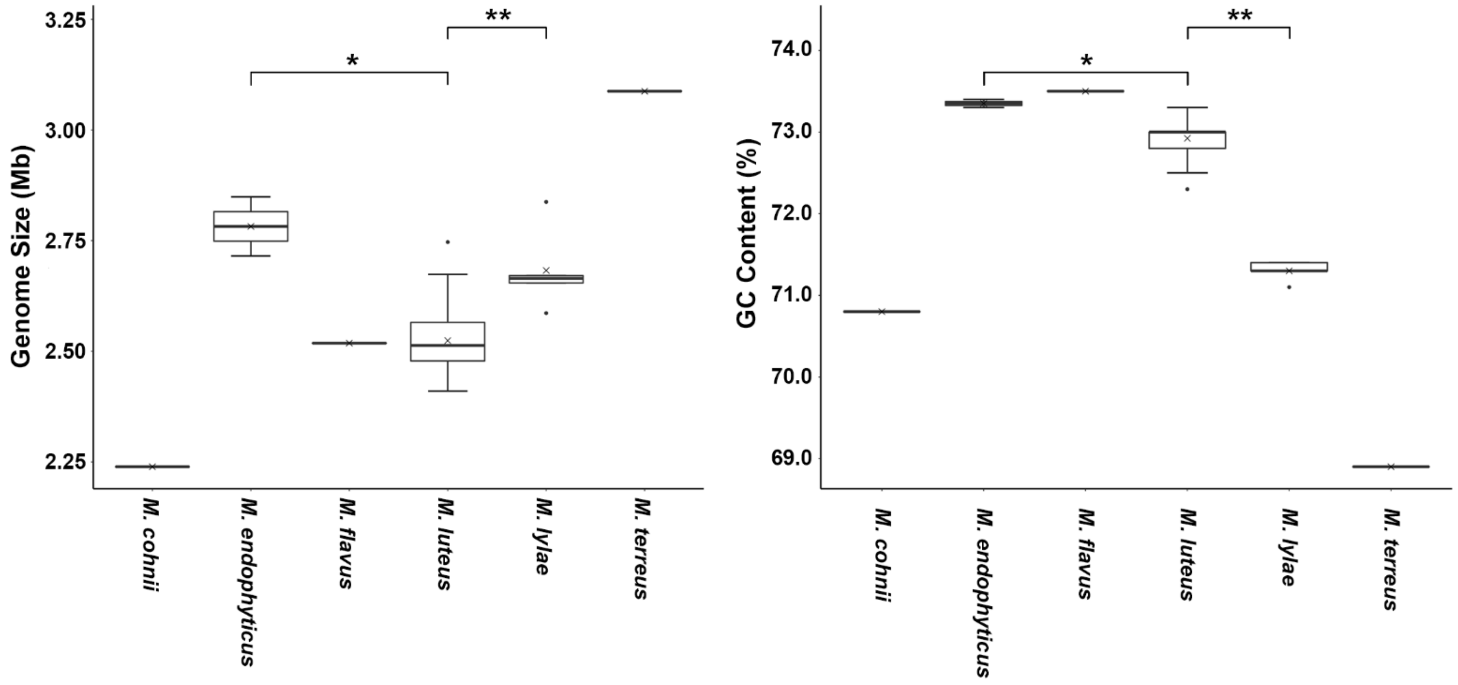

Figure S1. Variations in genome size and GC content between species in genus *Micrococcus*. Significant levels in variations were determined using Wilcoxon test (*, $P < 0.05$; **, $P < 0.01$).

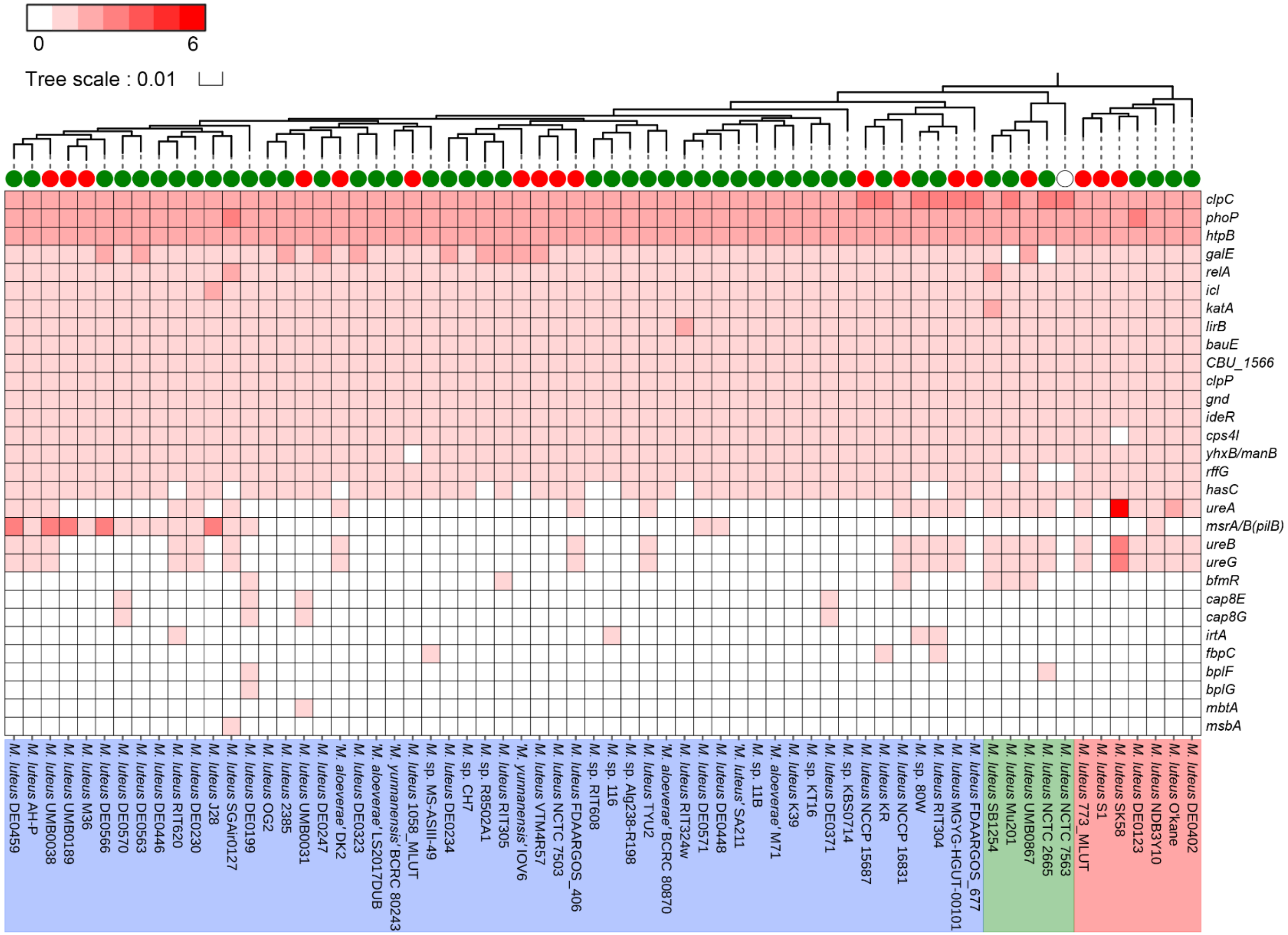

Figure S2. Heatmap of the distributions of the putative VF genes in *M. luteus* genomes. The color bar shows the number of genes. The tree topology is derived from FIG 3. Three major clades are presented by different background colors. The scale bar indicates 1% sequence divergence. Different habitats are represented by circles in different colors (red, HA; green, NHA; blank, uncertain).

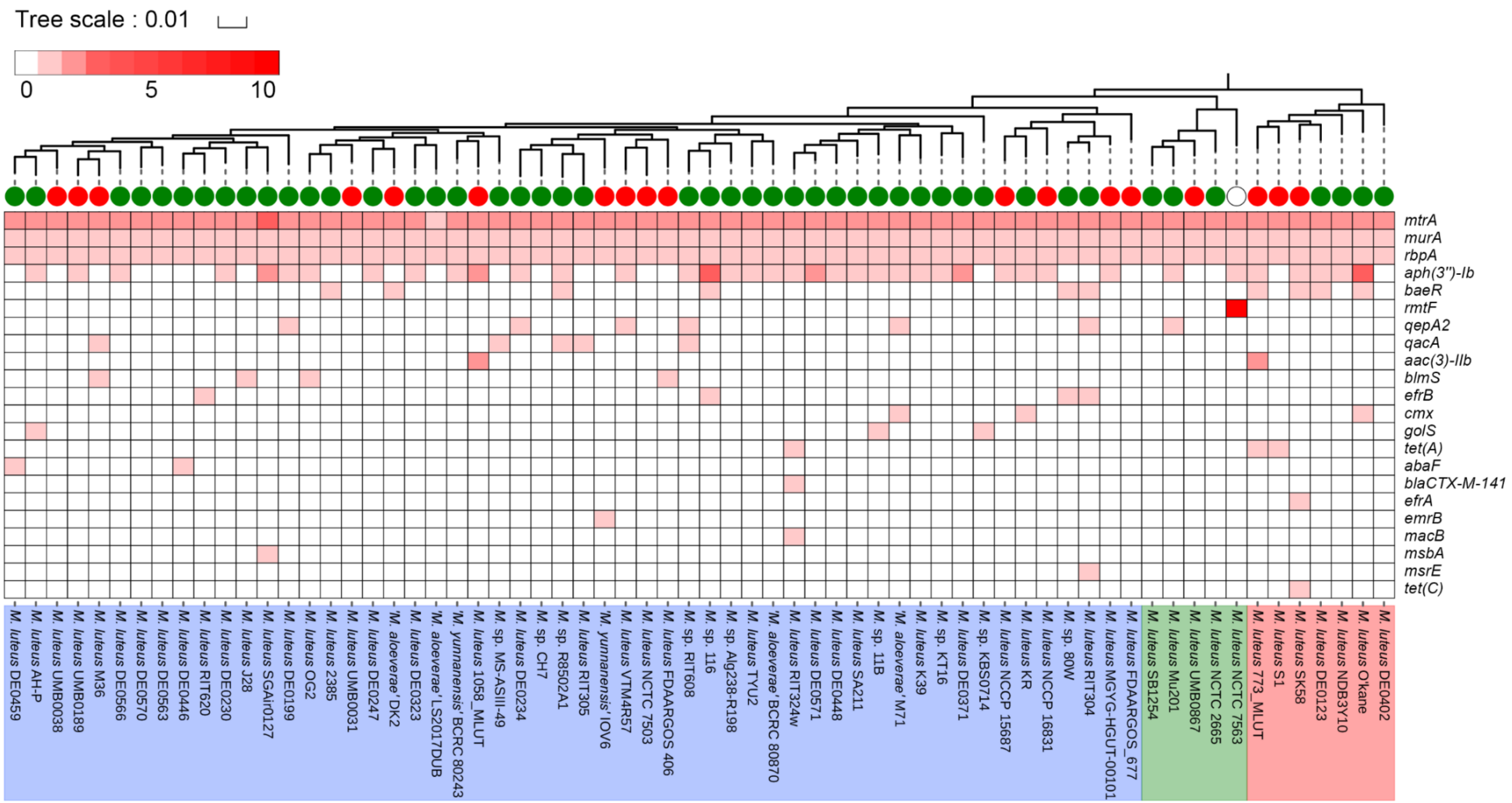

Figure S3. Antibiotic resistance gene profiles of *M. luteus* strains. The color bar shows the number of genes. The tree topology is derived from FIG 3. Three major clades are presented by different background colors. The scale bar indicates 1% sequence divergence. Different habitats are represented by circles in different colors (red, HA; green, NHA; blank, uncertain).

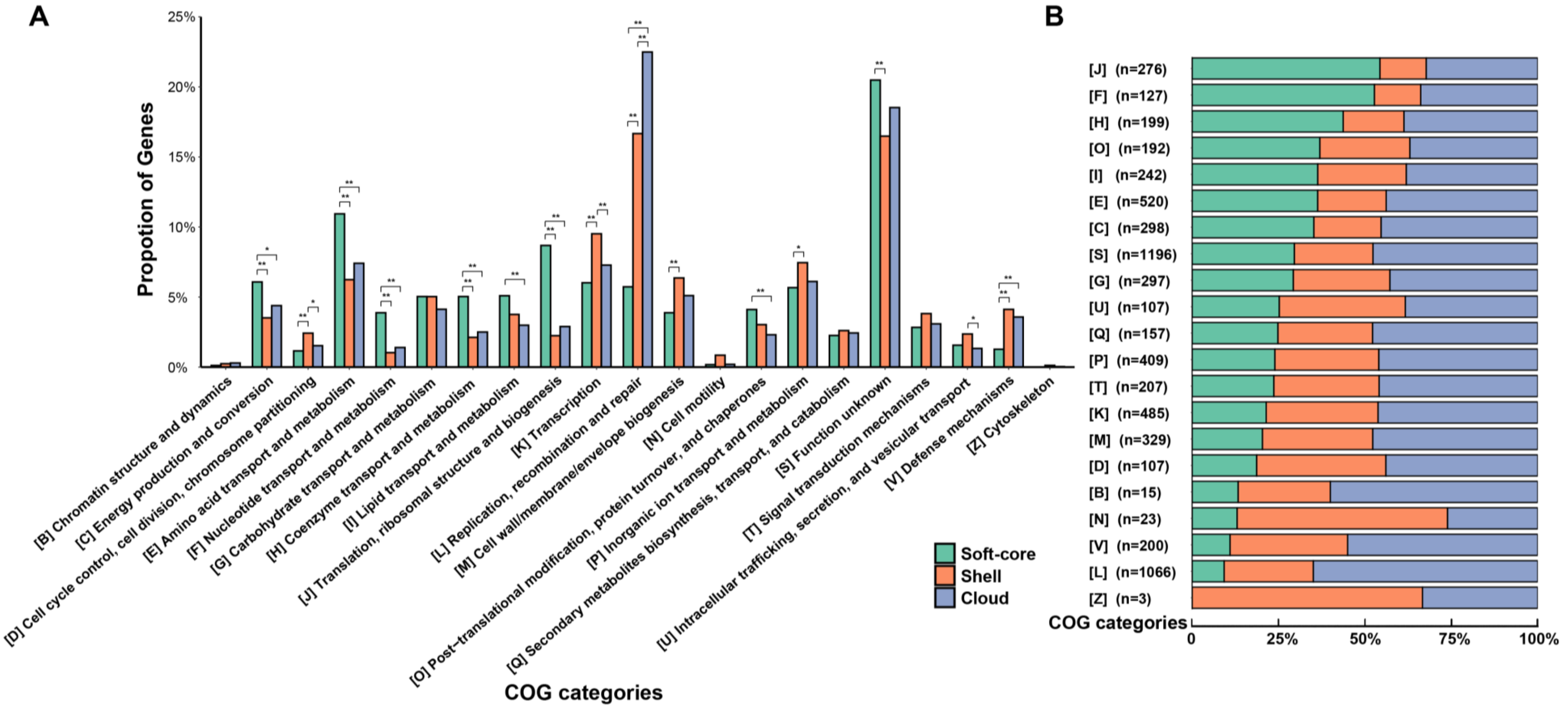

Figure S4. Comparison of the soft-core, shell and cloud genomes of *M. luteus* based on COG categories. Soft-core, shell and cloud genes were derived from GET_HOMOLOGUES. (A) Distribution of OGs in the soft-core, shell and cloud genomes. Asterisks indicate significant differences in proportions based on Fisher's exact test (*, $P < 0.05$; **, $P < 0.01$). (B) Partitioning of soft-core, shell and cloud genes for each COG category.

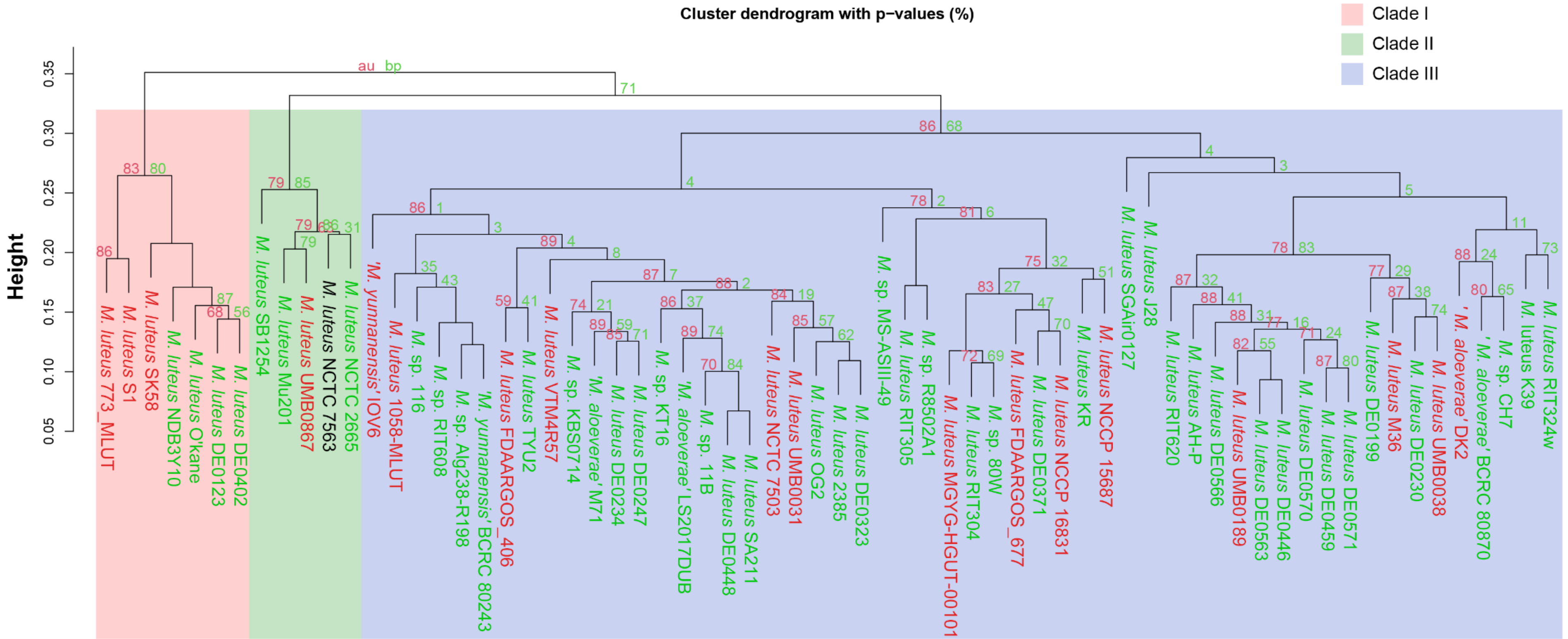

Figure S5. Hierarchical cluster analysis based on the presence or absence of dispensable genes. Two bootstrap resampling methods from 1,000 replicates were employed to assess the uncertainty in the hierarchical cluster analysis: approximately unbiased (au) in red and bootstrap probability (bp) in green. Only values lower than 90% are shown. Height indicates the dissimilarity between genomes. Three major clades are presented by different background colors. Strains collected from different habitats are represented in different colors (red, HA; green, NHA; black, uncertain).

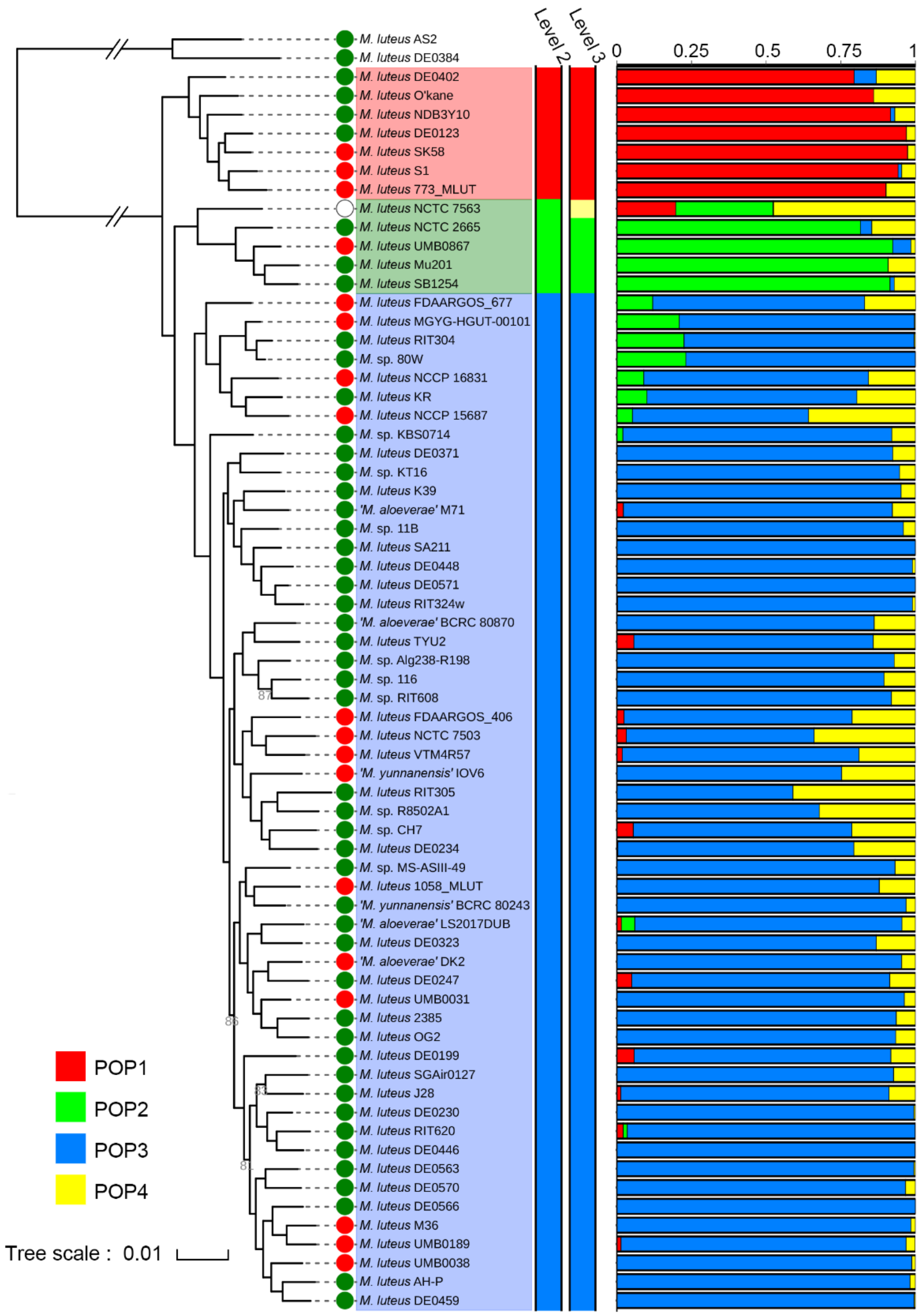

Figure S6. Population structure of *M. luteus*. Two levels of Fastbaps clusters and Structure plot of the 66 *M. luteus* strains are shown. Stacked bar chart shows the contribution to each *M. luteus* strain from each of the four hypothetical ancestral populations. The tree topology is derived from FIG 3. Three major clades are presented by different background colors. The scale bar indicates 1% sequence divergence. Strains from different habitats are represented by circles in different colors (red, HA; green, NHA; blank, uncertain).

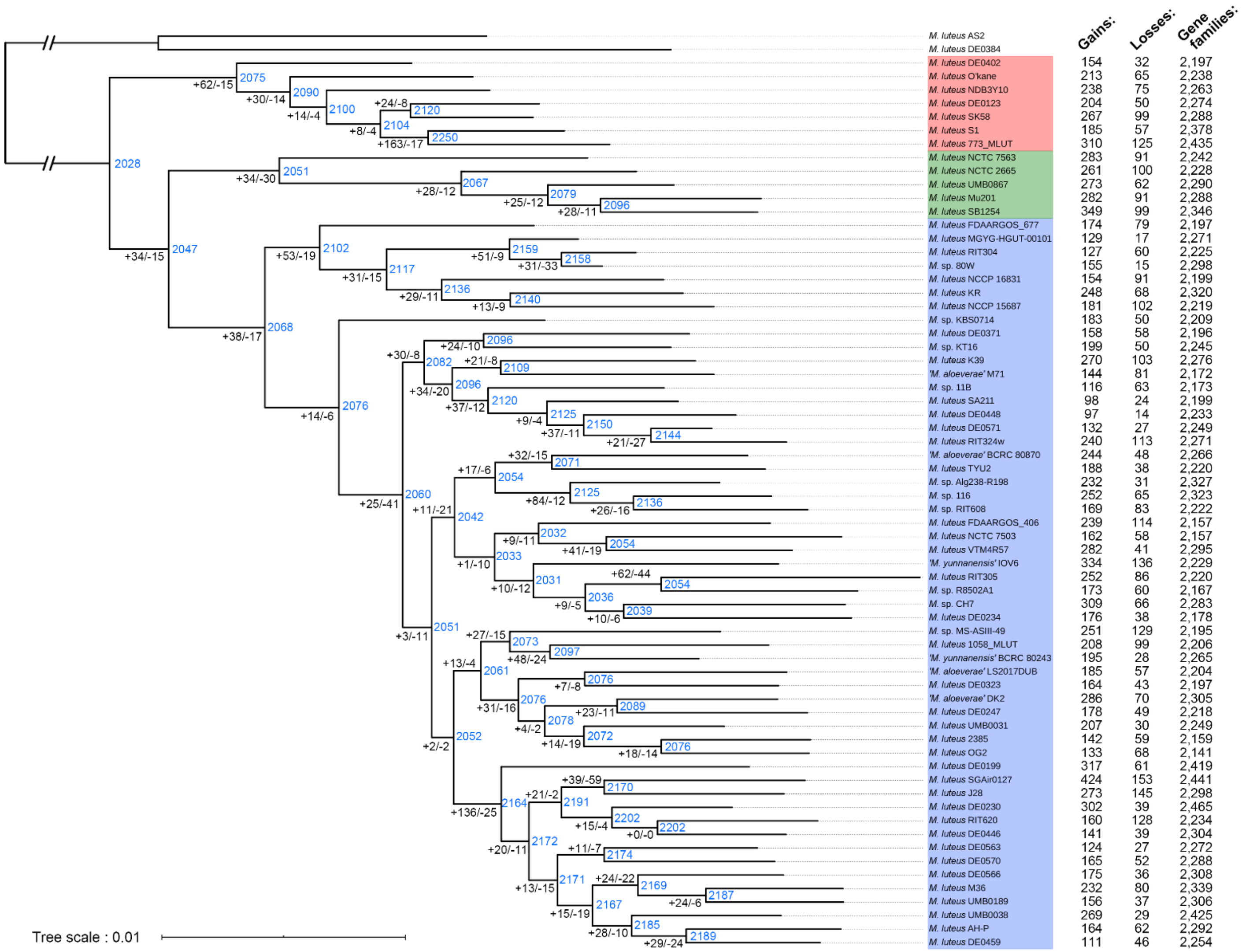

Figure S7. Ancestral genome content reconstruction using COUNT software. The tree topology is derived from FIG 3. The numbers of gain and loss events are shown at each lineage of the tree. “+”s represent gain events and “–”s represent loss events. The numbers in blue represent the total numbers of genes at nodes. Three major clades are presented by different background colors. The scale bar indicates 1% sequence divergence.
